# From Antarctica or Asia? New colonization scenario for Australian-New Guinean narrow mouth toads suggested from the findings on a mysterious genus *Gastrophrynoides*

**DOI:** 10.1186/1471-2148-11-175

**Published:** 2011-06-21

**Authors:** Atsushi Kurabayashi, Masafumi Matsui, Daicus M Belabut, Hoi-Sen Yong, Norhayati Ahmad, Ahmad Sudin, Mitsuru Kuramoto, Amir Hamidy, Masayuki Sumida

**Affiliations:** 1 Institute for Amphibian Biology, Graduate School of Science, Hiroshima University, Hiroshima, 739-8526, Japan; 2 Graduate School of Human and Environmental Studies, Kyoto University, Kyoto 606-8501, Japan; 3 Institute of Biological Sciences, Faculty of Science, University of Malaya, Kuala Lumpur, 50603, Malaysia; 4 Institute for Environment and Development (LESTARI), Universiti Kebangsaan Malaysia, UKM Bangi, Selangor, 43600, Malaysia; 5 Faculty of Science and Technology, Universiti Kebangsaan Malaysia, UKM Bangi, Selangor, 43600, Malaysia; 6 Institute for Tropical Biology and Conservation, University Malaysia Sabah, Kota Kinabalu, Sabah, 88999, Malaysia; 7 3-6-15 Hikarigaoka, Munakata, Fukuoka, 811-3403, Japan; 8 Museum Zoologicum Bogoriense, Research Center for Biology, Indonesian Institute of Sciences, Gedung Widyasatwaloka, Jalan Raya Jakarta Bogor km 46, Cibinong, West Java, Indonesia

## Abstract

**Background:**

Microhylidae is a geographically widespread family of anurans. Although several extensive molecular analyses have attempted to elucidate their subfamilial relationships, and correlate these with Mesozoic and Cenozoic continental drifts, consensus has not been reached. Further, generic level relationships have not been well investigated in some microhylid subfamilies, and therefore subfamilial affiliations of some genera are still unclear. To elucidate the phylogenetic positions of two mysterious Asian genera, *Gastrophrynoides *and *Phrynella*, and to better understand the trans-continental distributions of microhylid taxa, we performed molecular phylogenetic and dating analyses using the largest molecular dataset applied to these taxa to date.

**Results:**

Six nuclear and two mitochondrial genes (approx. 8 kbp) were sequenced from 22 microhylid frog species representing eight subfamilies. The maximum likelihood and Bayesian analyses could not fully elucidate the subfamilial relationships, suggesting a rapid radiation of these taxa between 85 and 66 million years ago. In contrast, generic relationships of Asian microhylines were generally well resolved.

**Conclusion:**

Our results clearly showed that one of two problematic Asian genera, *Phrynella*, was nested in the clade of the Asian subfamily Microhylinae. By contrast, *Gastrophrynoides *occupied the most basal position of the Australian-New Guinean subfamily Asterophryinae. The estimated divergence of *Gastrophrynoides *from other asterophryine was unexpectedly around 48 million years ago. Although a colonization scenario via Antarctica to the Australian-New Guinean landmass has been suggested for Asterophryinae, our finding suggested a novel colonization route via Indo-Eurasia.

## Background

Microhylidae is a large anuran family containing 487 species equivalent to 8% of all frogs [1]. This family belongs to the phylogenetically-nested anuran group, Neobatrachia, and forms Ranoides with Afrobatrachia (including the families, Arthroleptidae, Brevicipitidae, Hemisotidae, and Hyperoliidae) and Natatanura (= Ranidae *sensu lato*).

Members of the Microhylidae occur in most continents and several large islands, i.e., Africa, Eurasia (not in the subcontinent of Europe), South and North America, Australia, New Guinea, and Madagascar. Since Frost et al. [1], the subfamilial classification of this family had been largely modified based on new findings from several molecular phylogenetic studies [2,3]. Consequently, eleven microhylid subfamilies are now recognized [4] and each subfamily generally occurs in one landmass area derived from the Gondwana supercontinent as follows: Asterophryinae (Australia-New Guinea); Cophylinae, Dyscophinae, and Scaphiophryninae (Madagascar); Gastrophryninae and Otophryninae (South and North America); Hoplophryninae and Phrynomerinae (Africa); and Kalophryninae, Melanobatrachinae, and Microhylinae (Asia). Despite comprehensive phylogenetic studies, the subfamilial relationships have not been well elucidated (see Additional file 1). Further, this family contains 12 genera for which subfamilial affiliations have not been investigated [4]. The majority of these genera occur in South America, but two taxa are distributed in Asia. These mysterious genera contain only one to three species and difficulty in collecting them has prevented herpetologists from using them in phylogenetic study. Recently, we succeeded in obtaining specimens of the problematic Asian genera, *Gastrophrynoides *and *Phrynella*. Originally, *Gastrophrynoides *was a monotypic genus but the specimen used here is a newly found species of this genus (*G. immaculatus*) [5].

Because of their transcontinental distribution, microhylids have been regarded as an attractive research target for biogeography studies. Since Savage [6], several biogeographic scenarios that incorporate the Plate tectonics theory and breaking up the Gondwanan landmass, have been proposed to explain the trans-continental distribution of anuran taxa including microhylids [3]. Two molecular phylogenetic and dating analyses that aimed to elucidate the higher phylogeny, divergence ages, and formation process of the transcontinental distribution of microhylid taxa were recently performed [2,3]. These studies that used different taxa and molecular data resulted in different relationships and divergence ages for microhylid subfamilies. Consequently, consensus on a biogeographic scenario to explain the microhylid distribution pattern has not been reached. Furthermore, although these studies proposed different colonization scenarios for many microhylid taxa, they agree on a similar Antarctic route scenario for the Australian-New Guinean taxon (Asterophryinae), as suggested in other vertebrate taxa distributed in Australia (e.g., marsupials, ratite bird, chelid turtles, and hyloid frogs [7]).

It is generally considered that employing long sequence data, and increased taxon sampling in molecular phylogenetic inference, can clarify problematic phylogenetic relationships [8-10]. Thus, in this study, we sequenced two mitochondrial (mt) and six nuclear genes (total 8 kbp) from 22 microhylid specimens comprising eight out of 11 microhylid subfamilies, to determine the phylogenetic positions of the two problematic Asian microhylid genera, and re-examine the phylogenetic relationships and divergence ages of microhylid subfamilies with the long sequence data and additional samples. Based on our finding for the phylogenetic position and divergence age of the genus *Gastrophrynoides*, we advance a novel colonizing scenario for the Australian-New Guinean microhylids.

## Results and discussion

### Molecular phylogenetic analyses

The 35 specimens analyzed in this study are shown in Table 1. Briefly, we used 22 microhylid specimens from eight out of 11 known subfamilies, four afrobatrachians, five natatanurans, two hyloids, and two archaeobatrachians. From these specimens, we sequenced two mt and six nuclear genes approx. 8 kbp in total.

**Table 1:** Specimens used in this study and accession numbers of resultant sequences

Species	**Subfamily **(Family)	Voucher	Accession numbers							
**Microhylidae**			***rag1***	***rag2***	***tyr***	***bdnf***	***cox1***	***cxcr4***	***ncx1***	***16S***

*Barygenys flavigularis*	Asterophryinae	IABHU 6597	AB611856	AB611857	AB611858	AB611859	AB611860	AY948800[33]	AY948845[33]	AY948767[33]
*Calluella guttulata*	Microhylinae	No voucher (pettrade)	AB611861	AB611862	AB611863	AB611864	EF396041[3]	EF017975[2]	EF018031[2]	DQ283144[1]
*Chaperina fusca*	Microhylinae	BORN 8478	AB611865	AB611866	AB611867	AB611868	AB611869	AB611870	AB611871	AB611872
*Cophixalus cryptotympanum*	Asterophryinae	IABHU 6602	AB611873	AB611874	AB611875	AB611876	AB611877	AB611878	AB611879	AB611880
*Ctenophryne geayi*	Gastrophryninae	No voucher (pettrade)	AB611881	AB611882	AB611883	AB611884	AB611885	AB611886	AB611887	AB611888
*Dyscophus guineti *	Dyscophinae	No voucher (pettrade)	AB611889	AB611890	AB611891	AB611892	AB611893	AB611894	AB611895	DQ283434[1]
*Gastrophryne olivacea*	Gastrophryninae	KUHE 33224	AB611896	AB611897	AB611898	AB611899	AB611900	EF017968[2]	EF018005[2]	DQ347338[34]
*Gastrophrynoides immaculatus*	Unknown	UKM HC 279	AB611901	AB611902	AB611903	AB611904	AB611905	AB611906	AB611907	AB611908
*Kalophrynus interlineatus*	Kalophryninae	No voucher (pettrade)	AB611909	AB611910	AB611911	AB611912	AB611913	AB611914	AB611915	AB611916
*Kalophrynus pleurostigma*	Kalophryninae	No voucher (pettrade)	AB611917	AB611918	AB611919	AB611920	AB611921	AY948776[33]	AY948811[33]	DQ283146[1]
*Kaloula taprobanica*	Microhylinae	KUHE 37252	AB611922	AB611923	AB611924	AB611925	AB611926	AY948772[33]	AY948807[33]	AF249057[35]
*Metaphrynella pollicaris*	Microhylinae	KUZ 21655	AB611927	AB611928	AB611929	AB611930	AB611931	AB611932	AB611933	AB611934
*Metaphrynella sundana*	Microhylinae	BORN 8191	AB611935	AB611936	AB611937	AB611938	AB611939	EF017973[2]	EF018029[2]	EF017954[2]
*Microhyla annectens*	Microhylinae	KUHE 52438	AB611940	AB611941	AB611942	AB611943	AB611944	AB611945	AB611946	AB611947
*Microhyla marmorata*	Microhylinae	KUHE 32455	AB611948	AB611949	AB611950	AB611951	AB611952	AB611953	AB611954	AB611955
*Microhyla okinavensis*	Microhylinae	IABHU living individual	AB611956	AB611957	AB611958	AB611959	AB303950[36]	AB611960	AB611961	AB303950[36]
*Micryletta inornata*	Microhylinae	KUHE 35133	AB611962	AB611963	AB611964	EF396022[3]	AB611965	AB611966	AB611967	AB611968
*Phrynella pulchra*	Unknown	UKM HC 820	AB611969	AB611970	AB611971	AB611972	AB611973	AB611974	AB611975	AB611976
*Phrynomantis microps*	Phrynomerinae	No voucher (pettrade)	AB611977	AB611978	AB611979	AB611980	AB611981	AB611982	AB611983	AB611984
*Plethodontohyla inguinalis*	Cophylinae	UADBA AK041208-001	AB611985	AB611986	AB611987	AB611988	AB611989	AB611990	AB611991	AB611992
*Ramanella montana*	Microhylinae	Not preserved	AB611993	AB611994	AB611995	AB611996	AB611997	AB611998	AB611999	AB612000
*Scaphiophryne madagascariensis*	Scaphiophryninae	No voucher (pettrade)	AB612001	AB612002	AB612003	AB612004	AB612005	AB612006	AB612007	AB612008

**Afrobatrachia**										

*Arthroleptis variabilis*	(Arthroleptidae)	ZFMK 68794	EF396073[3]	EF396112[3]	AY341756[37]	AB612009	AB612010	AY364180[38]	AB612011	AB612012
*Hemisus marmoratus*	(Hemisotidae)	No voucher (pettrade)	AB612013	EF396127[3]	EF395975[3]	AB612014	AB612015	AY364186[38]	AY948827[33]	AB612016
*Hyperolius viridiflavus*	(Hyperoliidae)	No voucher (pettrade)	AY323769[39]	AY323789[39]	AB612017	EF396013[3]	AB612018	AB612019	AB612020	AB612021
*Trichobatrachus robustus *	(Arthroleptidae)	No voucher (pettrade)	EF396109[3]	AB612022	AY844192[40]	EF396035[3]	AB612023	AB612024	AB612025	AB612026

**Natatanura**										

*Blommersia wittei *	(Mantellidae)	ZSM (D48/2000)	AY323774[39]	AY323795[39]	AY341751[37]	AY323774[39]	AB612027	AB612028	AB612029	AB612030
*Buergeria buergeri*	(Rhacophoridae)	IABHU living individual	AB612031	AB612032	AB612033	AB612034	AB127977[41]	AB612035	AB612036	AB127977[41]
*Lithobates catesbeianus*	(Ranidae)	IABHU living individual	AB612037	AB612038	AB612039	AB612040	AB511303[42]	AB612041	AB612042	X12841[43]
*Mantella madagascariensis*	(Mantellidae)	IABHU 6933	AB612043	AB612044	AB612045	AB612046	AB212225[44]	AB612047	AB612048	AB212225[44]
*Staurois latopalmatus*	(Ranidae)	BORN 8098	AB612049	AB612050	AB612051	AB612052	AB511311[42]	EF017987[2]	EF018011[2]	AB511310[42]

**Hyloides**										

*Agalychnis callidryas*	(Hylidae)	No voucher (pettrade)	AY323765[39]	AY323780[39]	DQ283018[1]	AY323765[39]	AB612053	AB612054	AB612055	AB612056
*Bufo japonicus*	(Bufonidae)	IABHU 4001	AB612057	AB612058	AB612059	AB612060	AB303363[36]	AB612061	AB612062	AB303363[36]

**Archaeobatrachia**										

*Megophrys nasuta*	(Megophryidae)	No voucher (pettrade)	AB612063	AB612064	AB612065	AB612066	AB612067	AB612068	AB612069	AB612070
*Scaphiopus holbrookii*	(Scaphiopodidae)	No voucher (pettrade)	AB612071	AB612072	AB612073	AB612074	AB612075	AB612076	AB612077	AB612078

Numbers in braces indicate the data from previous studies listed in References. BORN: BORNEENSIS Collection, Universiti Malaysia Sabah; IABHU: Institute for Amphibian Biology, Hiroshima University; KUHE: Graduate School of Human and Environmental Studies, Kyoto University; KUZ: Department of Zoology, Kyoto University; UADBA: Université d'Antananarivo, Département de Biologie Animale; UKM HC: Universiti Kebangsaan Malaysia, Herpetological Collection; ZFMK: Zoologisches Forschungsmuseum Alexander Koenig; ZSM: Zoologische Staatssammlung München.

**Figure 1:**
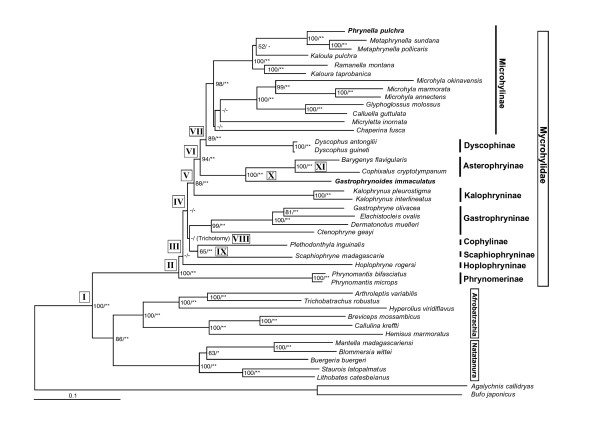
**Phylogenetic relationships of microhylids**. The ML tree (-ln*L *= 77832.21) based on the Aln-1 dataset (7164 nucleotide sites from two mitochondrial and six nuclear genes) is shown. Bootstrap probabilities of ML analysis (> 50%) and Bayesian post probabilities (* > 95, ** > 99%) are shown for each node. "Trichotomy" indicates the node condition in the corresponding BI trees. The nodes of which split ages are discussed in the text are shown by roman numerals (I - XI) and the same node numbers are used in Table 3.

Adding our data to that from two previous studies [2,3], we produced the longest aligned dataset (Aln-1, 7164 nucleotide sites) so far used with these taxa (see Methods section). Maximum likelihood (ML) and Bayesian interference (BI) analyses were performed on this dataset. The resultant ML tree is shown in Figure 1. The BI tree recovered an identical topology, except that one branch that was resolved in the ML tree collapsed to a trichotomy in the BI tree (see Figure 1). Our ML and BI trees strongly supported the monophyly of the family Microhylidae, the monophyly of each microhylid subfamily (*sensu *after Frost et al. [1]), and the generic relationships within each subfamily (with the exception of several microhyline genera, see below). Unfortunately, these trees could not fully elucidate subfamilial relationships (see below).

### Relationships of microhylid subfamilies

The two most basal nodes among the microhylids, Phrynomerinae and Hoplophryninae, are both African in distribution. The remaining subfamilies are divided into two clades. One clade consists of two Asian, one Australian-New Guinean, and one Madagascan taxa, i.e., Kalophryninae + (Asterophryinae + (Dyscophinae + Microhylinae)). The subfamilial relationships within this clade were well supported by bootstrap probabilities (BPs = 84-94%) and Bayesian posterior probabilities (BPPs = 100%). The other clade includes one American and two Madagascan taxa, Gastrophryninae + (Cophylinae + Scaphiophryninae). The relationships within this clade and among the basal African taxa were not strongly supported by either BP or BPP values.

Many alternative relationships have been suggested for these poorly-supported groups [1-3, and see Additional file 1]. We evaluated these alternatives using the likelihood based Approximately Unbiased (AU) and Kishino-Hasegawa (KH) tests and could not reject four of ten alternative relationships for the African, American, and Madagascan taxa (topologies 2-11 in Table 2). Considering the similar likelihood scores among our ML tree and the alternative topologies (topologies 1, 3-5, and 11 in Table 2), the lack of statistical difference may be due to low phylogenetic signal in the DNA sequences due to ancient rapid divergences of these microhylid taxa (85 - 66 Ma; see below).

**Table 2:** Comparison of log-likelihood differences between alternative topologies and results of KH and AU tests

No	Alternative topologies	Reference	Difference of log-likelihood value from ML tree and rejection of KH and AU tests		
			**Δln*L***	**KH**	**AU**

1	Topology of the ML tree from Aln-1 (-ln*L = *77832.21)	This study (= Fig. 1)	0.00	**-**	**-**
**Phrynomerinae **(African taxon)					
2	2 nd basal position of Phrynomerinae (Kalophryninae is most basal)	[1]	23.44	**+**	**+**
3	Most basal of Phrynomerinae + Gastrophryninae clade	[3]	8.11	**-**	**-**
**Hoplophryninae **(African taxon)					
4	Hoplophryninae + (Cophylinae + Scaphiophryninae) clade	[3]	4.80	**-**	**-**
5	Hoplophryninae + Cophylinae clade	[1]	7.88	**-**	**-**
6	Hoplophryninae + Asterophryinae clade	[3]	36.61	**++**	**++**
**Cophylinae and Scaphiophryne **(Madagascan taxa)					
7	3 rd basal position of Scaphiophryninae & Cophylinae + Kalophryninae clade	[2]	19.72	**+**	**+**
8	Scaphiophryninae + Microhylinae clade & Cophylinae + Hoplophryninae clade	[1]	87.37	**++**	**++**
**Gastrophryninae **(American taxon)					
9	4 th basal position of Gastrophryninae	[2]	19.72	**+**	**+**
10	Gastrophryninae + (Cophylinae + Hoplophryninae) clade	[1]	87.37	**++**	**++**
11	2 nd basal position of Gastrophryninae	[3]	4.43	**-**	**-**
**Kalophryninae **(Asian taxon)					
12	Most basal of Kalophryninae	[1]	23.44	**+**	**+**
13	Kalophryninae + Cophylinae clade	[2]	19.72	**+**	**+**
14	Kalophryninae+(Cophylinae + Scaphiophryninae) clade	[3]	15.79	**-**	**-**
**Asterophryinae, Dyscophinae, & Microhylinae **(Australian-New Guinean, Madagascan, Asian taxa)					
15	Asterophryinae + Dyscophinae clade & Microhylidae + Scaphiophryninae clade	[1]	80.83	**++**	**++**
16	Microhylidae + Dyscophinae clade & (Asterophryinae + Kalophryninae) + Hoplophryninae clade	[3]	44.43	**++**	**++**
***Gastrophrynoides***					
17	*Gastrophrynoides *+ (Asterophryinae + (Dyscophinae + Microhylinae))	This study*	168.78	**++**	**++**

ML and lnL refer maximum likelihood and log-likelihood value. ΔlnL indicates difference of log-likelihood value from that of the ML topology. For KH and AU tests, P > 0.05, < 0.05, and < 0.01 are shown by -, +, ++, respectively. *The Maximum likelihood tree under the constraint of "Gastrophrynoides is not monophyly with Asterophryinae".

In our trees, the Kalophryninae + (Asterophryinae + (Dyscophinae + Microhylinae)) clade seems to be well resolved. For these taxa, our dataset (Aln-1) was able to reject most alternative relationships proposed in previous studies (topologies 12-16 in Table 2). However, an alternative Kalophryninae position suggested by van der Meijden et al. [3] could not be rejected (topology 14 in Table 2). Furthermore, when we used a data subset (Aln-3) that contained a member of the subfamily Melanobatrachinae not present in the Aln-1 dataset, a different Kalophryninae position, Melanobatrachinae + (Cophylinae + Kalophryninae)), was recovered (Additional file 1). This suggests that the melanobatrachini data affected the Kalophryninae position. In contrast to the Kalophryninae case, the Asterophryinae + (Dyscophinae + Microhylinae) clade was well supported by two data subsets having different taxon samplings (Aln-2 and 3; Additional File 1). Two recent molecular phylogenetic studies also suggested this clade and the relationships within [3,4]. Thus, the monophyly of these Australian-New Guinean, Madagascan, and Asian taxa seems to be well established.

### Phylogenetic positions of mysterious microhylid genera

In this study, two Asian genera, *Phrynella *and *Gastrophrynoides*, of which subfamilial affiliations have not been investigated, were analyzed. Our ML and BI trees resulted in the genus *Phrynella *being nested in the Asian subfamily Microhylinae (Figure 1). By contrast, the genus *Gastrophrynoides *did not become a member of the Asian group, rather this taxon possessed the most basal position of the members of the Australasian-New Guinean subfamily, Asterophryinae (ML BP and BPP = 100%). The AU and KH tests clearly rejected the "non-monophyly of *Gastrophrynoides *and asterophryines" (*P *< 0.01; topology 17 in Table 2). Furthermore, the most basal position of *Gastrophrynoides *among asterophryines was also supported by our data subsets (Aln-2 and 3; ML BPs = 100% and BPPs = 100%, see Additional file 1). Consequently, our analyses clearly elucidated the phylogenetic positions of these problematic Asian genera. Around the same time as this study, Matsui et al. [11] suggested the *Phrynella *position within Microhylinae and a close relationship of *Gastrophrynoides *with Asterophryinae from their analyses using *12S *and *16S *gene data.

The synapomorphy of the subfamily Asterophryinae is direct developing eggs [1]. Although the breeding ecology of the genus *Gastrophrynoides *is not known, the pigment-less eggs and rudimentary webbings in *G. borneensis *[12] suggest direct development. Thus, we transiently regard this genus as a member of the subfamily Asterophryinae. Furthermore, the distribution of another asterophryine genus, *Oreophryne*, in islands of South-East Asia (e.g., Philippines, Sulawesi, and Bali) has been noted [4,13]. However, asterophryine species have not been reported from mainland Eurasia. Thus, *G. immaculatus *from the Malay Peninsula is the first record of the occurrence of a species belonging to the asterophryine lineage in mainland Eurasia.

### Generic relationships within Microhylinae

This study contains the first molecular phylogenetic analysis that covers all known microhyline genera (in Aln-3 data subset, see Additional file 1). Our analyses largely elucidated the phylogenetic relationships of microhyline genera, excluding the positions of *Kaloula *spp., *Chaperina*, and *Micryletta *(see below). The ML and BI trees from the Aln-1 dataset obtained the two major clades for microhyline genera with good statistical support: the *Microhyla *+ (*Calluella *+ *Glyphoglossus*) clade and the clade including *Kaloula, Metaphrynella, Phrynella*, and *Ramanella *(Figure 1). In the latter clade, the monophyly of *Metaphrynella *and *Phrynella *was strongly suggested (ML BP and BPP = 100%). Our data subsets also supported this clade (Additional file 1). Thus, the precise phylogenetic position of the genus *Phrynella *within the subfamily Microhylinae is clearly elucidated.

It is noteworthy that our analyses suggested the polyphyly of the genus *Kaloula *(Figure 1). The AU and KH tests clearly rejected the monophyly of this genus (data not shown). Our data subsets and the analyses of Van Bocxlaer et al. [2] also suggested that *Kaloula *as currently delimited is not a natural group (Additional file 1). Because *K. pulchra *(one of the two *Kaloula *species used here) is the type species of the genus *Kaloula*, the generic name of *K. taprobanica *might be altered in a future study.

### Divergence times of microhylid taxa

Using two data subsets (Aln-2d and Aln-3d) for which we had greater taxon sampling and our ML tree topology (see Additional file 2), we estimated divergence times. Three distinct combinations of calibration points were applied for each dataset, for a total six dating calculations (A-F). The estimated divergence times from these calibrations are summarized in Table 3, and the detailed results are shown in Additional file 3.

**Table 3:** Estimated divergence ages of microhylid taxa

		**Estimated divergence age (Ma) ± SD **[Min - Max values of 95% confidence interval]								
		
Node	Divergence events	Calibration A	Calibration B	Calibration C	Calibration D	Calibration E	Calibration F	**Average **(MD) **of calibration A-F**	Van Bocxlaer et al.[2]*1	Van der Meijden et al.[3]
		(from the Aln-2d + Tree1a combination)			(from the Aln-3d + Tree1b combination)					
I	**Split of microhylid lineage from other ranoids**	**143.2 ± 12.5** [121.6-170.3]	**134.6 ± 15.6** [106.4-167.3]	**130.0 ± 14.2** [103.8-159.3]	**136.6 ± 10.3** [118.1-158.6]	**133.0 ± 10.0** [115.3-154.4]	**118.5 ± 11.5** [98.0-142.8]	**132.6 **(9.6)	**127.3 ± 9.7** [109.9 - 147.9]	**116 ± 17** [87 - 153]

II	**Initial divergence of living microhylid subfamilies (Split of Phrynomerinae)**	**90.3 ± 8.5** [77.6-110.6]	**82.3 ± 11.8** [61.6-107.6]	**78.9 ± 10.6** [59.9-101.4]	**92.3 ± 7.1** [80.3-108.0]	**89.3 ± 6.8** [78.0-104.5]	**75.4 ± 9.3** [58.7-95.1]	**84.8 **(8.4)	**88.0 ± 6.5** [77.0 - 102.2]	66 ± 11*^3^ [47 - 90]

III	**2 nd basal split of microhylid subfamilies (Split of Hoplophryninae)**	**88.7 ± 8.4** [76.3-108.6]	**80.7 ± 11.6** [60.3-105.7]	**77.4 ± 10.5** [58.7-99.6]	**87.7 ± 6.4** [77.1-102.1]	**84.6 ± 6.1** [74.7-98.6]	**70.9 ± 8.8** [55.1-89.6]	**81.7 **(8.2)	**83.9 ± 5.9** [74.2 - 97.1]	NS (**57 - 66**)*^4^

IV	**Split of Gastrophryninae + (Cophylinae + Scaphiophryninae) clade from other microhylids**	**85.8 ± 8.0** [74.2-105.1]	**78.0 ± 11.3** [58.2-102.2]	**74.8 ± 10.2** [56.6-96.4]	**83.8 ± 5.8** [74.3-97.1]	**80.8 ± 5.6** [72.0-93.8]	**67.3 ± 8.5** [52.2-85.3]	**78.4 **(8.1)	NA	NA

V	**Split of Kalophryninae from Asterophryinae + (Dyscophinae + Microhylinae) clade**	**81.4 ± 7.7** [70.7-100.1]	**73.9 ± 10.8** [54.8-97.1]	**70.9 ± 9.8** [53.4-91.6]	**81.8 ± 5.6** [72.7-94.5]	**78.8 ± 5.3** [70.5-91.3]	**65.3 ± 8.3** [50.5-83.0]	**75.3 **(7.9)	76.8 ± 5.0*^2^ [68.9 - 88.7]	NA

VI	**Split of Asterophryinae from Dyscophinae + Microhylinae clade**	**76.6 ± 7.1** [67.2-94.2]	**69.3 ± 10.3** [51.2-91.3]	**66.5 ± 9.3** [49.9-86.2]	**76.8 ± 5.0** [68.9-88.6]	**73.9 ± 4.9** [66.9-85.7]	**60.8 ± 7.9** [46.8-77.7]	**70.6 **(7.7)	**73.6 ± 4.5** [66.7 - 84.5]	**57 ± 10** [40 - 79]

VII	**Split of Dyscophinae and Microhylinae**	**73.6 ± 6.8** [65.3-90.7]	**66.5 ± 10.0** [49.0-87.9]	**63.7 ± 9.0** [47.7-83.0]	**72.6 ± 4.4** [66.4-83.3]	**69.7 ± 4.2** [65.1-80.6]	**56.8 ± 7.5** [43.5-72.9]	**67.2 **(7.6)	**68.7 ± 3.4** [65.1 - 77.6]	**55 ± 10** [39 - 76]

VIII	**Split of Gastrophryninae from Cophylinae + Scaphiophryninae clade**	**83.9 ± 8.0** [72.1-103.0]	**76.3 ± 11.1** [56.7-100.2]	**73.1 ± 10.0** [55.1-94.3]	**80.4 ± 6.0** [70.1-93.8]	**77.6 ± 5.8** [67.8-90.7]	**64.7 ± 8.3** [49.8-82.3]	**76.0 **(7.9)	NA	NA

IX	**Split of Cophylinae and Scaphiophryninae**	**72.1 ± 7.8** [59.8-90.0]	**65.4 ± 10.1** [47.8-87.3]	**62.7 ± 9.2** [46.4-82.3]	**71.2 ± 6.8** [59.0-85.4]	**68.9 ± 6.6** [57.1-83.0]	**57.5 ± 8.1** [43.1-74.8]	**66.3 **(6.8)	NA	**53 ± 9** [38 - 74]

X	**Split of *Gastrophrynoides *from other asterophryines**	**53.7 ± 6.5** [43.1-68.5]	**48.5 ± 8.2** [34.3-66.2]	**46.5 ± 7.5** [33.4-62.6]	**50.1 ± 5.5** [40.3-61.7]	**48.4 ± 5.3** [39.0-59.8]	**39.7 ± 6.3** [28.7-53.3]	**47.8 **(5.4)	NA	NA

XI	**Initial divergence of non-*Gastrophrynoides *asterophryines**	**22.7 ± 4.3** [15.5-32.3]	**20.5 ± 4.6** [12.9-30.7]	**19.7 ± 4.2** [12.6-29.1]	**30.4 ± 4.3** [22.8-39.6]	**29.4 ± 4.2** [22.0-38.6]	**24.1 ± 4.5** [16.5-33.8]	**24.5 **(5.4)	**26.8 ± 4.0** [19.8 - 35.3]	**20 ± 5** [12 - 30]

Node, roman numerals corresponding to Fig. 1. MD, mean difference. NA, not applicable. NS, not shown by the authors. *^1^They used two distinct dating methods and many distinct calibration point settings. The ages listed here were from the Bayesian molecular clock method and a calibration point setting (without point G) used as main result in their paper. *^2^Corresponding to the split age between Cophylinae + (Kalophryninae + Melanobatrachinae) clade and Asterophryinae + (Dyscophinae + Microhylinae) clade in [2]. *^3^Corresponding to the split age of Phrynomerinae + Gastrophryninae clade from other microhylids in [3]. *^4^Corresponding to the split of Otophryninae from other microhylids occurred between II and VI.

Similar divergence ages of microhylid taxa were estimated from the six calibrations; for most microhylid nodes, the median age estimated from one calibration was overlapped by the 95% confidence interval (CI) values from the other calibrations. The exceptions were several node ages that differed between the calibrations A and F. The average ages (and mean difference = MD) of the six calibrations for major microhylid divergence events are as follows (Table 3). (I) 132.6 Ma (MD = 9.6) for the split of microhylids from other ranoids. (II) 84.8 Ma (MD = 8.4) for the initial divergence of extant microhylid subfamilies (i.e., split of Phrynomerinae from other microhylids). (III) 81.7 Ma (MD = 8.2) for the divergence of Hoplophryninae from the remaining microhylid subfamilies. (IV) 78.4 Ma (MD = 8.1) for split of the Gastrophryninae + (Cophylinae + Scaphiophryninae) lineage. (V) 75.3 Ma (MD = 7.9) for the split of Kalophryninae from the Asterophryinae + (Dyscophinae + Microhylinae) clade. (VI) 70.6 Ma (MD = 7.7) for the splits of Asterophryinae from the Dyscophinae + Microhylinae clade. (VII) 67.2 Ma (MD = 7.6) for the split of Dyscophinae and Microhylinae. (VIII) 76.0 Ma (MD = 7.9) for the split of Gastrophryninae from the Cophylinae + Scaphiophryninae clade. (IX) 66.3 Ma (MD = 6.8) for the split of Cophylinae and Scaphiophryninae. (X) 47.8 Ma (MD = 5.4) for the split of *Gastrophrynoides *from asterophryine genera. (XI) 24.5 Ma (MD = 5.4) for the initial divergence of asterophryine genera (excluding *Gastrophrynoides*).

Additional calibrations (G-J) based on two alternative topologies (the ML topologies inferred from the Aln-2 and Aln-3 data subsets) also estimated similar ages for these microhylid divergences (Additional files 4 and 5). Two previous molecular dating studies (Table 3) have focused on these taxa. Our divergence times are similar to those from Van Bocxlaer et al. [2], but are slightly older than those from van der Meijden et al. [3].

Based on our estimations, the microhylid subfamilies diverged between 85 and 66 Ma (nodes II-IX in Table 3 and Figure 1). These estimated ages suggest a rapid radiation of major microhylid lineages, within less than 20 Ma during the late Cretaceous. According to traditional Plate-Tectonic theory, Gondwanan landmasses (Africa, South America, Madagascar-India, and Australia) had already fragmented (≈ 100 Ma [e.g., 14]) by this time. Thus, although several vicariance hypotheses based on Gondwanan fragmentation have been proposed for the trans-continental distribution of microhylids [see 3], our results can definitively reject a strictly vicariant scenario. Rather, our estimated dates fit a dispersal hypotheses (including overseas dispersal) [3] and/or the prolonged existence of land connections among the fragmented Gondwana landmasses [2].

### Colonization route of Australian-New Guinean taxa

This study could not resolve relationships among many microhylid subfamilies. However, the clade of Madagascan Dyscophinae + Asian Microhylidae and the sister relationship of Australian-New Guinean Asterophryinae with this clade are well established. Furthermore, we revealed that the genus *Gastrophrynoides*, which is only found in areas derived from the Eurasian landmass (Borneo and the Malay Peninsula), occupies the most basal phylogenetic position among asterophryines, and this taxon split from other asterophryine lineages during the Eocene (around 48 Ma).

**Figure 2:**
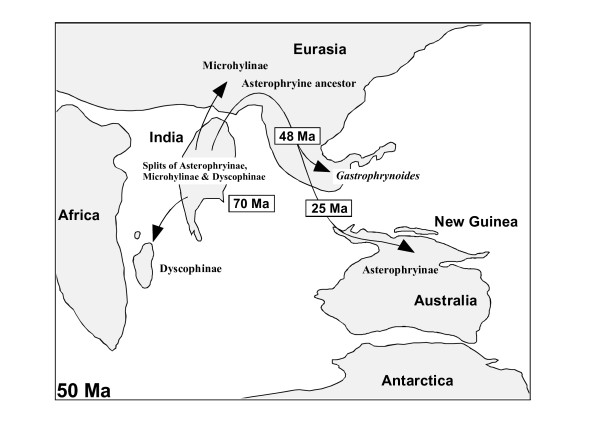
**Possible colonization route for asterophryine microhylids**. A colonization route hypothesis for Asterophryinae suggested from this study is shown on a schematic paleogeographic map (around 50 Ma).

An Antarctic route (across a land bridge existing until 55 Ma or less [e.g., 13]) has been postulated by two independent studies [2,3] as the colonization route of Asterophryinae into the Australian-New Guinean landmass. However, our new phylogenetic placement of the genus *Gastrophrynoides *and the estimated divergence times of *Gastrophrynoides *from its related taxa seem to suggest an alternative colonization pathway, from Asia to the New Guinean landmass, for Asterophryinae (Figure 2). In this context, the lineage split between Asterophryinae and Microhylinae (and Dyscophinae) occurred in the Indian landmass during the late Cretaceous (around 70 Ma), and these ancestors colonized Asia by the collision of India and Eurasia. The lineages of *Gastrophrynoides *and other asterophryines split during the Eocene (around 48 Ma, the same time as the date of the collision). Then, the ancestor of major asterophryines moved from Asia to the Australian-New Guinean landmass via islands and/or short sea straits around the late Oligocene (25 Ma) when both landmasses had been closing, and Southeast Asian islands had been uplifting [15]. If the ancestor had acquired the direct development characteristics, the synapomorphy of asterophryines, which can eliminate the use of a freshwater environment for the egg and tadpole development, oversea dispersal of this ancestor would have occurred relatively easily [16]. Finally, radiation of asterophryines mainly occurred in the New Guinean area and several lineages moved to Australia.

Compared with the Antarctic route, our proposed scenario accounts well for the very low species diversity of asterophryines in Australia (19 species, only 7% of all asterophryines [13]) relative to that on New Guinea and for the recent divergence time of asterophryines (< 25 Ma), both of which are difficult to explain in the Antarctic scenario [3]. Furthermore, to explain the placement of *Gastrophrynoides *under the Antarctic scenario, one would have to assume long-distance overseas dispersal of the *Gastrophrynoides *lineage from Australia to Asia during the Eocene, when the Southeast Asian islands had not yet formed, followed by the extinction of basal asterophrynine taxa (including the *Gastrophrynoides *lineage) only in Australian and New Guinean areas.

A weakness of our Asian route scenario is a lack of confidence in the sister taxon of the Asterophryinae + (Dyscophinae + Microhylinae) clade, the limited taxon sampling of asterophryines in our analyses, and the absence of other basal taxa belonging to the asterophryine lineage in other Asian areas, especially in India. Thus, to support our hypothesis, further phylogenetic studies, ideally supplemented by the discovery of new fossils, and more comprehensive surveys for living microhylids in Asian areas will be necessary. Undoubtedly, the discovery of the unique taxon *Gastrophrynoides *from Asian area has revealed a new concept for the asterophryine evolution. Further this finding has significance to show the possible presence of further microhylid taxa with unexpected evolutionary backgrounds and give a basis for future paleontological and biogeographic studies of Asian anurans.

## Conclusions

In this study, we performed phylogenetic analyses for higher microhylid taxa with the largest molecular data so far applied. Our results clearly indicate that one of two problematic Asian genera, *Phrynella*, is a member of the Asian subfamily Microhylinae. By contrast, *Gastrophrynoides *possesses the most basal position of the Australian-New Guinean subfamily Asterophryinae (Figure 1), and it is estimated that *Gastrophrynoides *split from other asterophryine occurred around 48 Ma (Table 3). The presence of the most basal asterophryine taxon in the Eurasian area suggests the colonization route from Asia to Australia for asterophryines (Figure 2), although a colonization scenario via Antarctica to the Australian-New Guinean landmass has been suggested for Asterophryinae. The biogeographic findings on *Gastrophrynoides *imply the possible occurrence of further microhylid taxa with unexpected evolutionary backgrounds and give a basis for future paleontological and biogeographic studies of Asian anurans.

## Methods

### Taxonomic names and frog specimens used

Since Frost et al. [1], taxonomic ranks and names of many anuran taxa including microhylids have changed frequently. To avoid needless confusion, in this paper, taxonomic ranks and names followed Frost et al. [1] and Frost [4], respectively. The 35 specimens analyzed here are shown in Table 1.

### PCR and sequencing

Total genomic DNA was extracted from muscle tissue of each specimen using a DNeasy Tissue Kit (QIAGEN) according to the manufacturer's protocol. From the total DNA, partial portions of two mt genes, 16S ribosomal RNA (*16S*, approx. 0.9 kbp) and Cytochrome c oxidase subunit I (*cox1*, approx. 0.8 kbp), and six nuclear encoding genes, brain-derived neurotrophic factor (*bdnf*, approx. 0.7 kbp), chemokine receptor 4 (*cxcr4*, approx. 0.7 kbp), Na^+^/Ca^2+ ^exchanger (*ncx1*, approx. 1.3 kbp), recombination-activating proteins 1 and 2 (*rag1 and rag2*, approx. 1.6 and 1.2 kbp, respectively) and tyrosinase (*tyr*, approx. 0.7 kbp), were amplified by PCR. These gene portions cover almost all sequence regions of the alignment data used in two previous molecular phylogenetic studies of microhylids (*16S, ncx1, cxcr4*, and *rag1 *[2]; *cox1, bdnf, tyr, rag1 *and *rag2 *[3]), and the amplification primers used here basically followed these studies. The detailed sequences of PCR and sequencing primers are available upon request (to AK). PCR mixtures were prepared with an Ex-Taq Kit (TaKaRa Bio) or a KOD-FX Kit (TOYOBO) according to the manufacturer's protocols. The resultant PCR fragments were purified by ExoSAP-It for PCR Clean-Up kit (US Biochemical) and ethanol precipitation. The gene sequences were directly determined from the purified PCR fragments with a BigDye^® ^Terminator cycle sequencing kit and an automated DNA sequencer (ABI3130xl, Applied Biosystems). The resultant sequence data were deposited in the DNA databases (Table 1).

### Molecular phylogenetic analyses

To perform phylogenetic analyses, we produced a long alignment dataset (Aln-1) and two data subsets (Aln-2 and Aln-3) by combining our data with that from Van Bocxlaer et al. and/or van der Meijden et al. [2,3] (Additional file 6). The long dataset (Aln-1) made from the data of all three studies includes a long sequence (7164 nucleotide sites in total) of eight gene partitions of 42 taxa (29 microhylids from nine subfamilies, six afrobatrachians, five natatanurans, and two hyloids). Among these two data subsets, the Aln-2 has a middle length sequence (4122 nucleotide sites) of five gene portions from 82 OTUs consisting of 53 microhylids from ten subfamilies, eight afrobatrachians, seven natatanurans and three hyloids, six archaeobatrachians, a caudate, and four other vertebrates. In Aln-2, *cox1 *sequences of non-neobatrachian taxa were not used because of the fast nucleotide substitution rate of this gene [3]. The Aln-3 data subset includes four gene portions (2813 nucleotide sites) from 63 taxa consisting of 44 microhylids from ten microhylid subfamilies, eight afrobatrachians, nine natatanurans, and two hyloids. More detailed information of used taxa and sequences in each alignment data are summarized in the Additional file 7. To make these alignments, we initially aligned each gene portion by using MUSCLE [17] implemented in SeaView ver. 3.2 [18]. The resultant alignments were revised by eye using amino acid alignments as the guide. For *16S *data, ambiguous alignment sites were removed by using Gblocks ver. 0.91 b [19] with a default parameter. Then, each gene portions were concatenated to make the above alignment datasets. The datasets used in this study are available from Additional file 8.

Based on the long dataset (Aln-1) and two data subsets (Aln-2 and 3), phylogenetic trees were constructed by the ML and BI methods. Heterozygous nucleotides occasionally found in nuclear gene sequences were deleted (and assign as missing data). Gaps in the alignments were treated as missing data. For ML and BI analyses, partitioned models were applied. The most appropriate substitution model for each gene portion was estimated based on the Akaike and Bayesian Information Criteria (AICc1 and BIC1 [20,21]) implemented in Kakusan3 [22] for ML and BI analyses, respectively. In ML analyses, the parameters for nucleotide frequencies, gamma distribution (G; with eight categories), and proportion of invariable sites were estimated by Treefinder program ver. Oct. 2008 [23]. The estimated best-fit models for each partition are shown in Additional file 6.

The ML analyses were performed using the Treefinder, and BP values were calculated with 1000 pseudo-replications. The BI analyses were performed using MrBayes ver. 3.1.2 [24]. Two independent runs of four Markov chains were conducted for 11 million generations for all datasets (sampling frequency was one tree per 100 generations for every datasets). Parameter estimates and convergence were checked with Tracer ver. 1.4 [25], and the first 1 million trees and first 3 million trees were discarded for Aln-1 and 2 data and Aln-3 data, respectively. Node credibility of the BI tree was evaluated by Bayesian posterior probabilities (BPP). Two hyloids (*Agalychnis callidryas *and *Bufo japonicus*) and zebrafish (*Danio rerio*) were employed as outgroups in the analyses based on Aln-1 and-3 data and Aln-2 data, respectively.

Our ML tree topology and alternative microhylid phylogenies suggested by previous studies [1-3] were compared in an ML framework using approximately unbiased (AU) and Kishino-Hasegawa (KH) tests [26,27] implemented in Treefinder. For the phylogenetic position of *Gastrophrynoides*, alternative topologies having high ln*L *scores were searched under the "non-monophyly of *Gastrophrynoides *and asterophryines" constraint. In this analysis, we used "are NOT" option of tree constraint command implemented in PAUP4.0 b [28]. Among the trees obtained under this constraint, one topology with the highest ln*L *score was also tested (topology 17 in Table 3).

### Molecular dating

Divergence time estimations using a Bayesian molecular clock method were conducted as in previous molecular dating analyses on microhylids [2,3]. We used two combinations of an alignment dataset and a topology, Aln-2d + Tree1a and Aln-3d + Tree1b (see below). Three sets of calibration points were applied to each combination (see below) for a total six dating analyses (calibration A-F). The topology and the applied calibration points in each calibration are shown in Additional file 2 and the alignment datasets used are available in Additional file 8.

The first alignment dataset used (Aln-2d) is basically the same with the Aln-2 but *cox1 *and *tyr *sequences were removed from the original Aln-2. This is because these genes are unsuitable for molecular dating of microhylid subfamilies (due to high nucleotide substitution rates [3]), and the *tyr *sequences did not allow us to calculate variance-covariance matrices of branch lengths for the designated topology (Tree1a), possibly due to many nodes lacking supporting nucleotide changes. Thus, Aln-2d only contains *rag-1, rag-2 *and *bdnf *sequences (2970 nucleotide sites in total). The other alignment data (Aln-3d) is similar to Aln-3 but this data contains a larger number of OTUs (total 101) to allow us to employ broad calibration points that were used in Van Bocxlaer et al. [2]. The Aln-3d (2739 sites in total) is slightly shorter than the original Aln-3 because of increment of ambiguous alignment sites due to adding OTUs. Zebrafish (*Danio rerio*) was employed as outgroup for both Aln-2d and-3d analyses. The two tree topologies (Tree1a and Tree1b) used in the age calibrations were modified from the ML trees of Aln-2 and Aln-3 datasets, respectively; they have the ML topology of Aln-1 (= Figure 1) for microhylids, natatanurans, and afrobatrachians (see Additional File 2), while all other relationships were as inferred for the respective datasets.

A total 14 calibration points, based on nine fossil records (F1-9) and five paleogeographic events (G1-5), were applied in this study as indicated below. F1: > 330 Ma, split of Lissamphibia and Amniota (fossil of the earliest aïstopod). F2: 338 - 312 Ma, split of Diapsida and Synapsida (fossils of early diapsids and synapsids). F3: > 230 Ma, split of Anura and Caudata (fossil of *Triadobatrachus*). F4: > 164 Ma, split of Costata (Alytidae and Bombinatoridae) from other anurans (fossil of *Eodiscoglossus*). F5: > 151 Ma, split of Rhinophrynidae and Pipidae (fossil of *Rhadinosteus*). F6: > 146 Ma, split of Cryptobranchidae and Hynobiidae (fossil of *Chunepeton*). F7: > 55 Ma, split of Bufonidae and other hyloid families (fossil of the oldest Bufonidae). F8: > 29 Ma, split of *Rana (sensu lato) *and other ranid genera (fossil of the oldest *Rana*). F9: > 404 Ma, split of lungfishes and tetrapods (fossil of the oldest tetrapodomorph [29]). G1: > 110 Ma, split of *Pipa *and *Xenopus *(fragmentation of the African and South American landmasses). G2: > 65 Ma, split of Dyscophinae and Microhylinae (fragmentation of India and Madagascar). G3: > 42 Ma, split of *Agalychnis *and *Litoria *(fragmentation of Australia and South America). G4: < 15 Ma, *Blommersia wittei *and *B*. sp. "Comoro" (formation of Comoro Islands). G5: > 5 Ma, *Alytes muletensis *and *A. dickhilleni *(the Mediterranean salinity crisis). With the exception of F9, these calibration points were applied in Van Bocxlaer et al. [2] and/or van der Meijden et al. [3]. The minimum ages of F1 and F2 were adjusted from those used by van der Meijden et al. (from 338 and 288 to 330 and 312, respectively) based on more recent information [30]. Also to accommodate more recent information, the minimum divergence times of F2 and F3 were changed from the original values used by Van Bocxlaer et al. (from 306.1 and 245.0 to 312 and 230, respectively).

Seven calibration points, (F1-F3, F8, and G1-G3) were applied in calibrations A and D. The seven points (F1-F3, G1, and G3-G5) previously used in van der Meijden et al. [3], plus F9, were applied in calibration B. Six points (F1-F3 and F7-F9) consisting of only fossil evidences were applied in calibration C. Nine points (F1-8 and G2) previously used in Van Bocxlaer et al. [2] were applied in calibration E. Eight fossil points (F1-8) were applied in calibration F.

The age calibrations were performed using software packages PAML ver. 4 [31] and Multidivtime [32]. In all calibrations, optimized branch lengths with their variance-covariance matrices of each alignment data were estimated for each gene partition (i.e., multiple loci analysis) with an F84 + G model (similar to the best model among available models in Multidivtime) using estbranches program. Parameters used in the model were estimated by PAML. To estimate divergence times, Markov chains were conducted for 10 million cycles with one per 100 sampling frequency and 10% burn-in for all six calibrations.

Four additional dating analyses (calibrations G-J) were performed using two distinct tree topologies (= the topologies of maximum likelihood trees from Aln-2 and Aln-3 datasets; see Additional file 4). The procedures of these calculations were much the same as the above but 3 million Markov chain cycles were conducted for these additional calculations.

## Authors' contributions

AK contributed to sample collection, molecular works, data analyses, drafting the manuscript, and design of this study. AH, DMB, MK, SM, and MM participated in the field works and improving the manuscript. H-SY, NA, and AS contributed to the sample collections. All authors read and approved the final manuscript.

## Supplementary Material

Additional file 1 **Phylogenetic trees from our data subsets and previous studies**. Two ML trees from our data subsets (Aln-2 and 3) and three phylogenetic trees from previous studies. Click here for file

Additional file 2 **Time trees from calibration A and D**. Time trees from the calibration A and D are shown. Click here for file

Additional file 3 **Estimated ages from calculations A-F**. Detailed estimated ages from the calibrations A-F are written in tabular form. Click here for file

Additional file 4 **Time trees from calibrations H and J**. Time trees from the calibration H and J are shown. Click here for file

Additional file 5 **Estimated ages from the calibrations G-J**. Detailed estimated ages from the calibrations A-F are written in tabular form. Click here for file

Additional file 6 **Information of sequence data and substitution models**. Data partitions and fitted nucleotide substitution model for each partition in the alignment datasets are shown. Click here for file

Additional file 7 **Information of OTUs and sequence accession numbers**. List of taxa used for this study and their sequence accession numbers. Click here for file

Additional file 8 **Alignment datasets**. All alignment datasets used (Aln-1, 2, 2 d, 3, and 3 d) are provided in nexus format. Click here for file
