# Supplementary material for: From Antarctica or Asia? New colonization scenario for Australian-New Guinean narrow mouth toads suggested from the findings on a mysterious genus Gastrophrynoides
Source: BMC Evol Biol. 2011 Jun 21;11:175. doi: 10.1186/1471-2148-11-175 (PMC3141433; doi:10.1186/1471-2148-11-175)
Supplement: Additional file 1 — Phylogenetic trees from our data subsets and previous studies. Two ML trees from our data subsets (Aln-2 and 3) and three phylogenetic trees from previous studies. [file 1471-2148-11-175-S1.PDF]

Additional file 1.

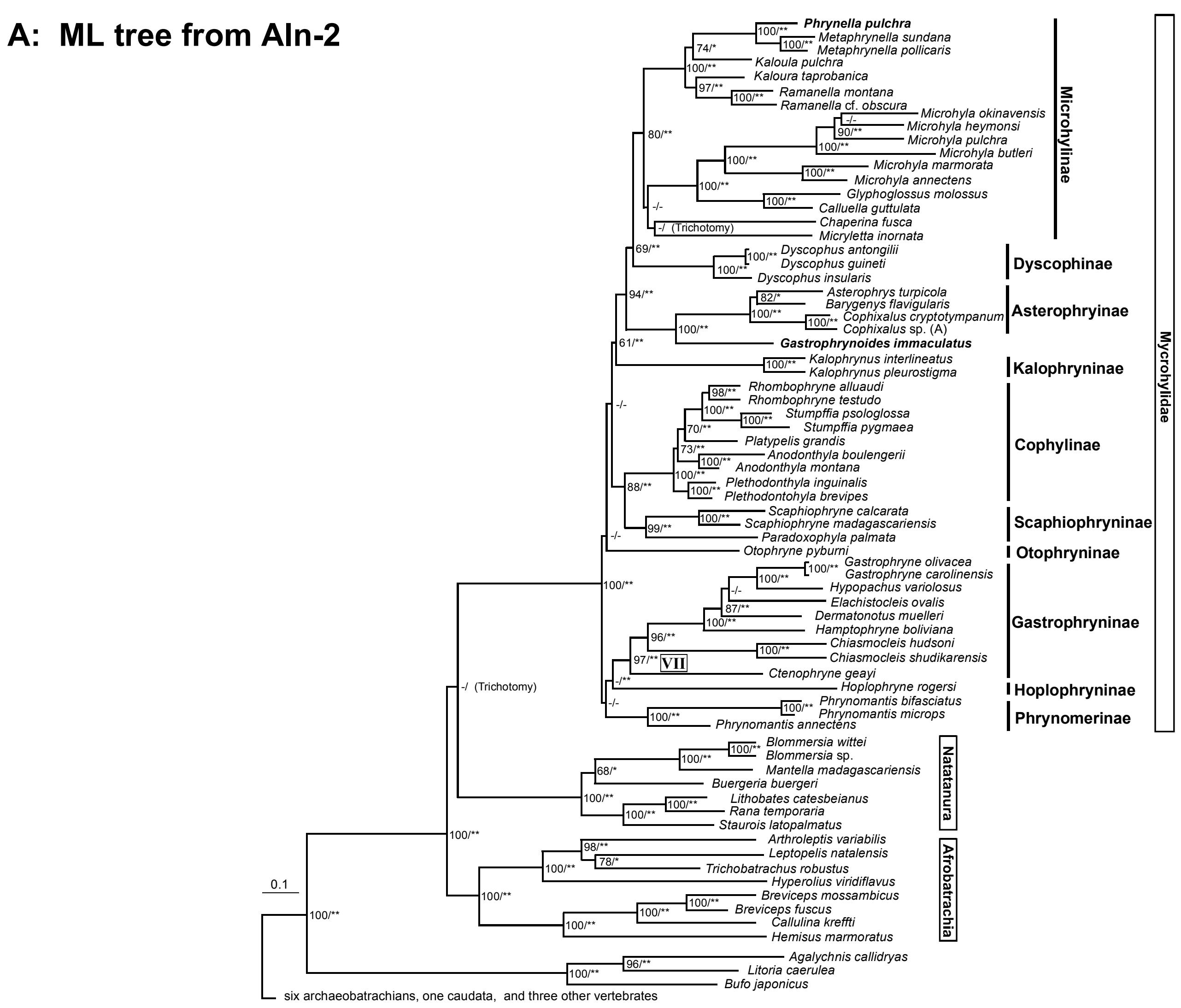

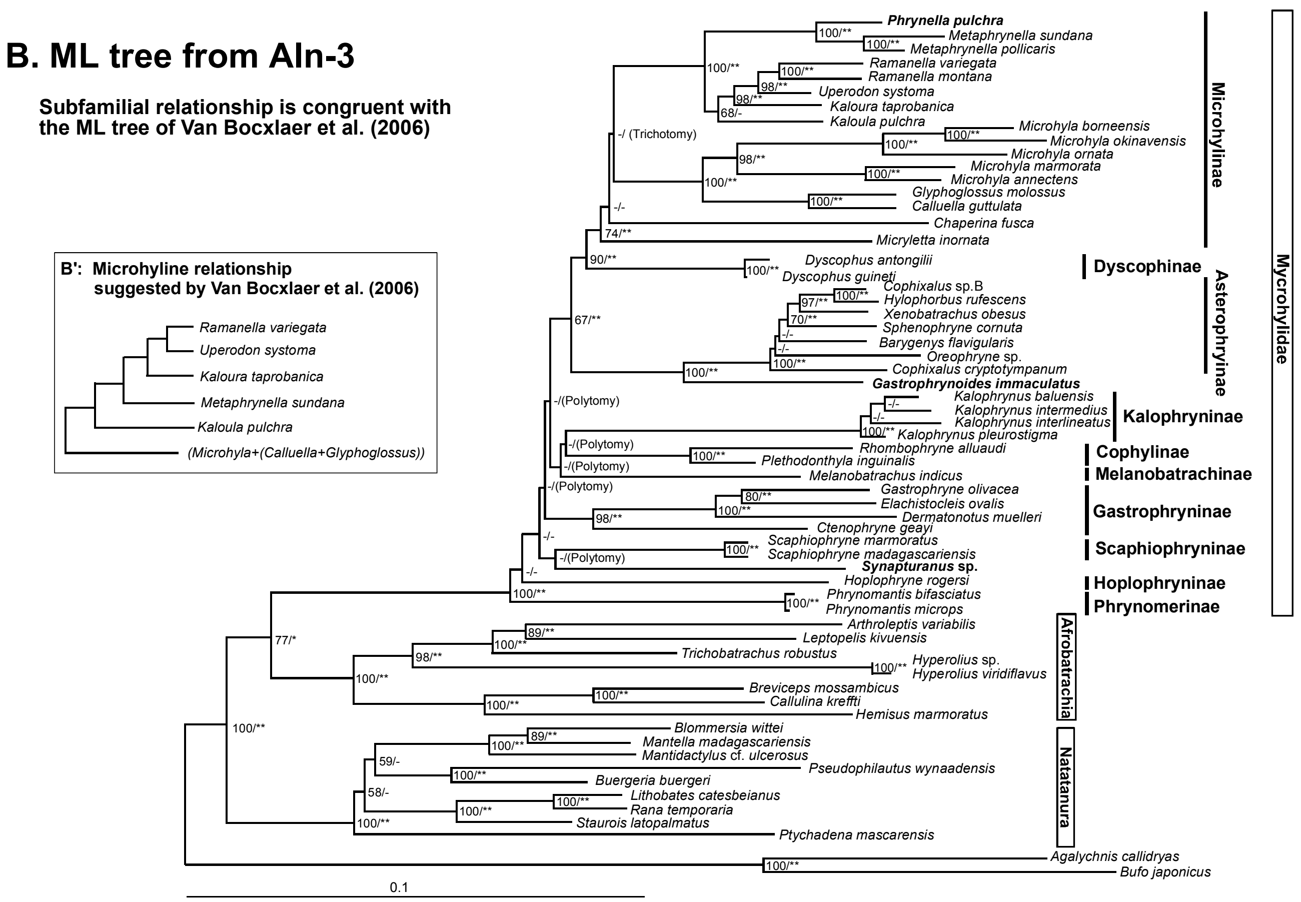

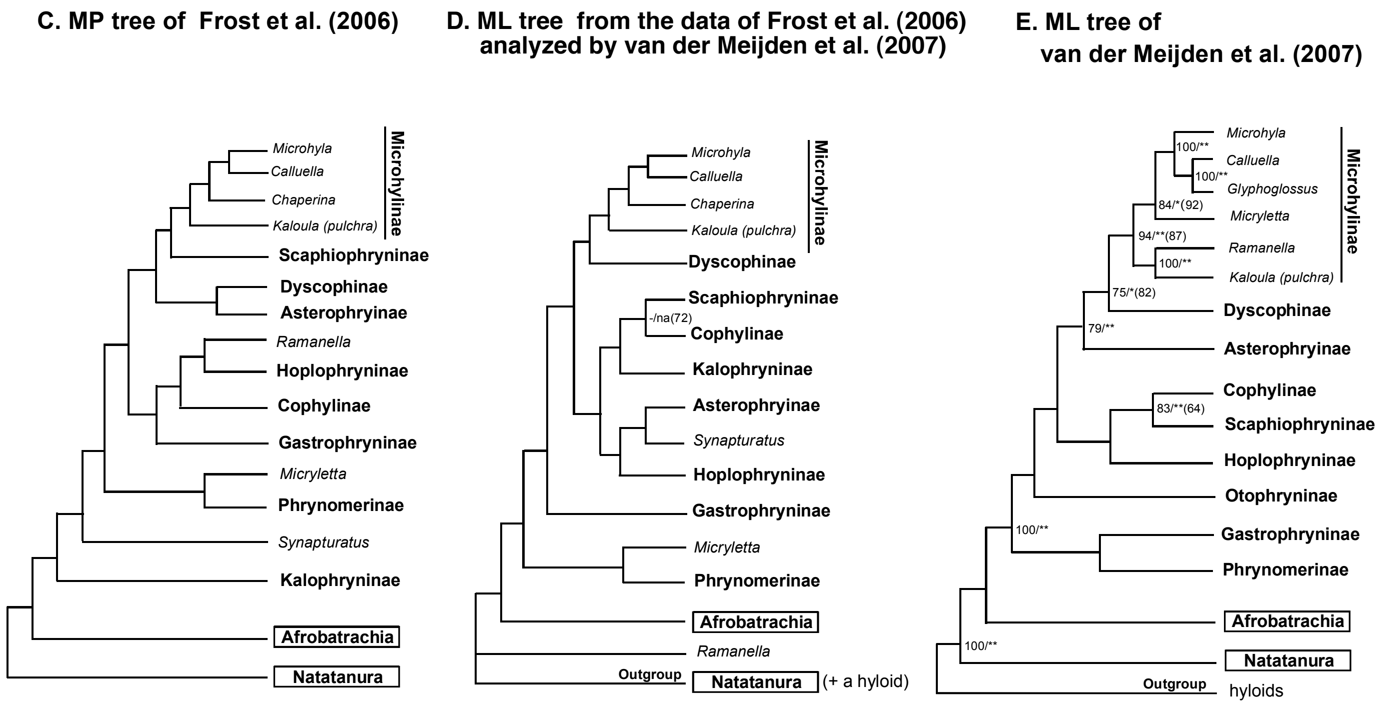

Additional file 1. Phylogenetic trees from our data subsets and previous studies.

A: ML tree based on the Aln-2 dataset (4122 nucleotide sites from one mitochondrial and four nuclear genes; -ln*L* = 84493.19). Non-ranoids are omitted in this figure. B: ML tree based on the Aln-3 dataset (2813 nucleotide sites from one mitochondrial and three nuclear genes; -lnL = 34679.14). The subfamilial relationship is congruent with that of the ML tree of Van Bocxlaer et al. [3]. B': Generic relationship of microhylines suggested by Van Bocxlaer et al. [3]. C: MP tree from Frost et al. [2]. D: ML tree from the data of Frost et al., analyzed by van der Meijden et al. [4]. E: ML tree from van der Meijden et al. [4]. Bootstrap probabilities of ML analyses and Bayesian post probabilities (* > 95, ** > 99) are shown for each node. “Different topology”, “Trichotomy”, and “Polytomy” in A and B indicate the node condition in the corresponding BI trees. In D and E, > 50 % bootstrap probabilities of the most parsimonious analyses are shown in parentheses.
