# Supplementary material for: From Antarctica or Asia? New colonization scenario for Australian-New Guinean narrow mouth toads suggested from the findings on a mysterious genus Gastrophrynoides
Source: BMC Evol Biol. 2011 Jun 21;11:175. doi: 10.1186/1471-2148-11-175 (PMC3141433; doi:10.1186/1471-2148-11-175)

Additional file 2. Time trees from calibrations A and D

Time tree from Calibration A

The topology shown here (Tree1a) was used in not only Calibration A but also Calibrations B and C.
Calibration points applied in the Calibrations A - C are shown.
Node numbers (1 ~ 80) correspond to those in Additional file 3.

Calibration point	Age constraint	Applied calibrations (and calibration point setting)
F1	> 330 Ma	Calibration A, B, C (Cps-Common, Meijden, Fossil1)
F2	338-312 Ma	Calibration A, B, C (Cps-Common, Meijden, Fossil1)
F3	> 230 Ma	Calibration A, B, C (Cps-Common, Meijden, Fossil1)
F7	> 55 Ma	Calibration C (Fossil1)
F8	> 29 Ma	Calibration A, C (Cps-Common, Fossil1)
F9	> 404 Ma	Calibration B, C (Cps-Meijden, Fossil1)
G1	> 110 Ma	Calibration A and B (Cps-Common and Meijden)
G2	> 65 Ma	Calibration A (Cps-Common)
G3	> 42 Ma	Calibration A and B (Cps-Common and Meijden)
G4	< 15 Ma	Calibration B (Cps-Meijden)
G5	> 5 Ma	Calibration B (Cps-Meijden)

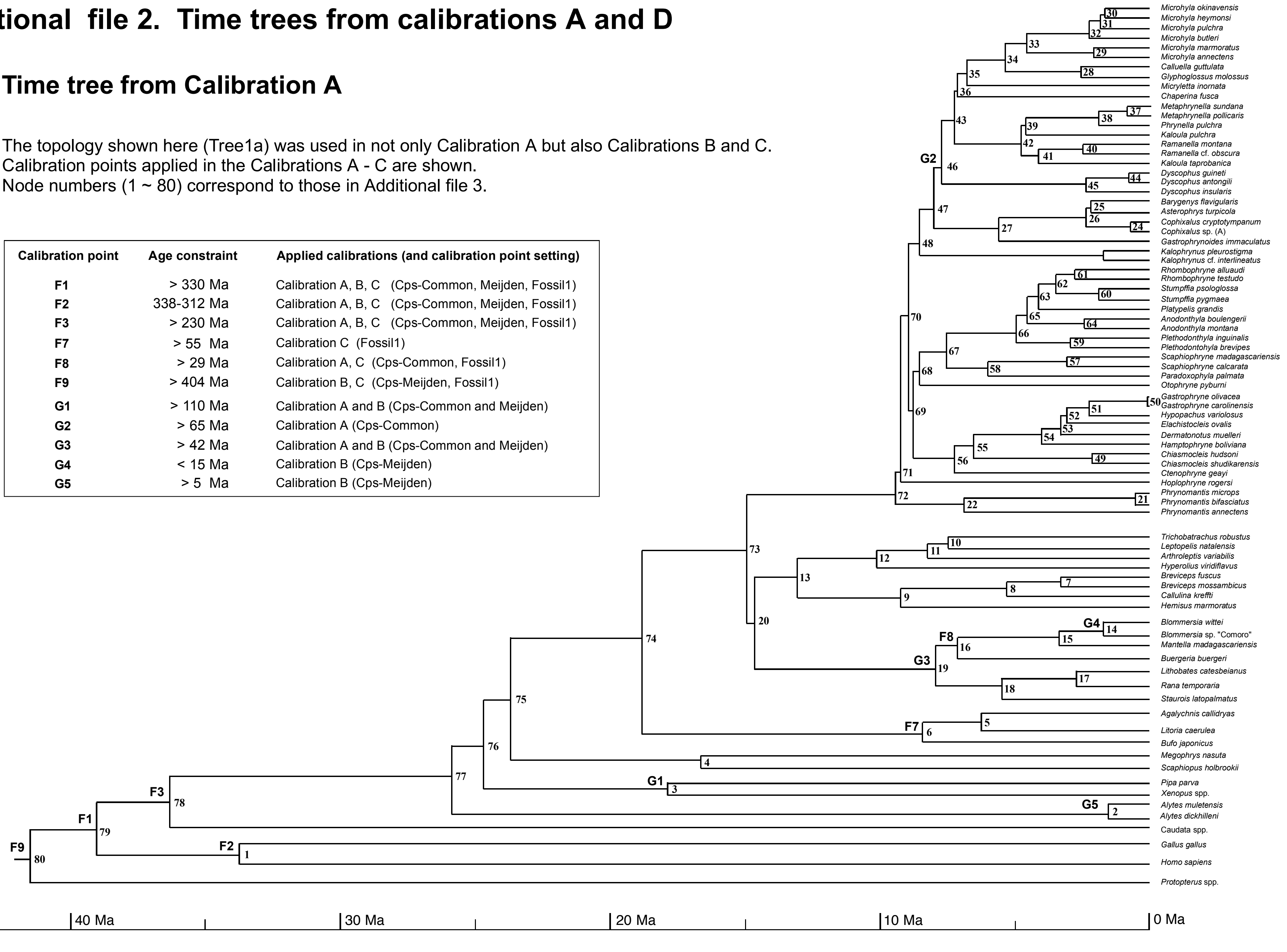

Time tree from Calibration D

The topology shown here (Tree1b) was used in not only Calibration D but also Calibrations E and F.
Calibration points applied in the Calibrations D - F are shown.
Node numbers (1 ~ 99) correspond to those in Additional file 3.

Calibration point	Age constraint	Applied calibration (Calibration point setting)
F1	> 330 Ma	Calibration D, E, F (Cps-Common, Bocxlaer, Fossil2)
F2	338-312 Ma	Calibration D, E, F (Cps-Common, Bocxlaer, Fossil2)
F3	> 230 Ma	Calibration D, E, F (Cps-Common, Bocxlaer, Fossil2)
F4	> 164 Ma	Calibration E, F (Cps-Bocxlaer, Fossil2)
F5	> 151 Ma	Calibration E, F (Cps-Bocxlaer, Fossil2)
F6	> 146 Ma	Calibration E, F (Cps-Bocxlaer, Fossil2)
F7	> 55 Ma	Calibration E, F (Cps-Bocxlaer, Fossil2)
F8	> 29 Ma	Calibration D, E, F (Cps-Common, Bocxlaer, Fossil2)
G1	> 110 Ma	Calibration D (Cps-Common)
G2	> 65 Ma	Calibration D, E (Cps-Common, Bocxlaer)
G3	> 42 Ma	Calibration D (Cps-Common)

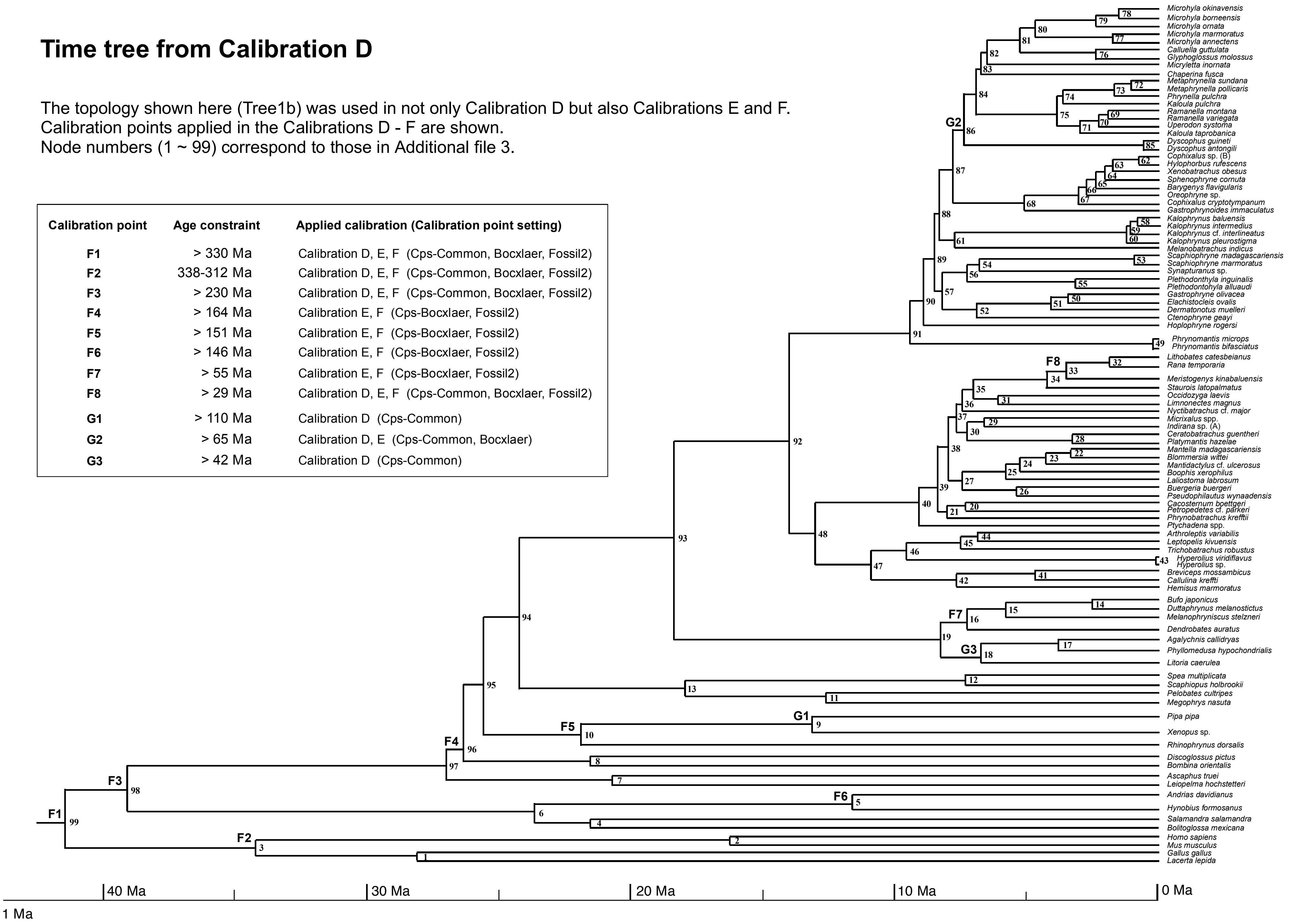

Supplement: Additional file 2 — Time trees from calibration A and D. Time trees from the calibration A and D are shown. [file 1471-2148-11-175-S2.PDF]
