# Supplementary material for: From Antarctica or Asia? New colonization scenario for Australian-New Guinean narrow mouth toads suggested from the findings on a mysterious genus Gastrophrynoides
Source: BMC Evol Biol. 2011 Jun 21;11:175. doi: 10.1186/1471-2148-11-175 (PMC3141433; doi:10.1186/1471-2148-11-175)

Additional file 4. Time trees from calibrations H and J

Time tree from calibration H

The topology shown here (ML tree topology from Aln-2 dataset) was used in Calibrations G and H.
Calibration points applied in these calibrations are shown.
Node numbers (1 ~ 80) correspond to those in Additional file 5.

Calibration point	Age constraint	Applied calibration(s)
F1	> 330 Ma	Calibrations G and H
F2	338-312 Ma	Calibrations G and H
F3	> 230 Ma	Calibrations G and H
F8	> 29 Ma	Calibration G
F9	> 404 Ma	Calibration H
G1	> 110 Ma	Calibrations G and H
G2	> 65 Ma	Calibration G
G3	> 42 Ma	Calibration G and H
G4	< 15 Ma	Calibration H
G5	> 5 Ma	Calibration H

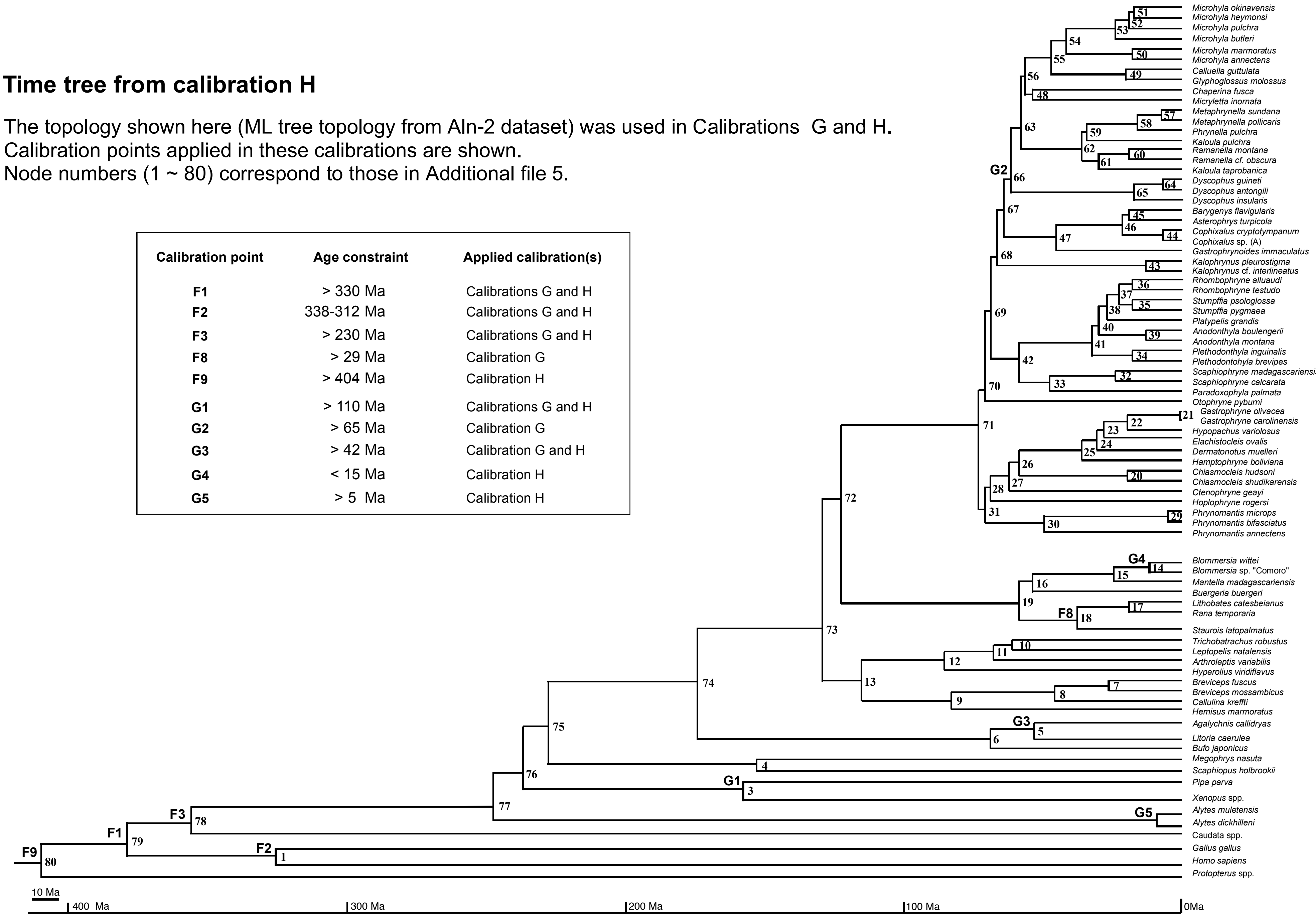

Time tree from calibration J

The topology shown here (ML tree topology from Aln-3 dataset) was used in Calibrations I and J.
Calibration points applied in these calibrations are shown.
Node numbers (1 ~ 99) correspond to those in Additional file 5.

Calibration point	Age constraint	Applied calibration(s)
F1	> 330 Ma	Calibrations I and J
F2	338-312 Ma	Calibrations I and J
F3	> 230 Ma	Calibrations I and J
F4	> 164 Ma	Calibration J
F5	> 151 Ma	Calibration J
F6	> 146 Ma	Calibration J
F7	> 55 Ma	Calibration J
F8	> 29 Ma	Calibrations I and J
G1	> 110 Ma	Calibration I
G2	> 65 Ma	Calibrations I and J
G3	> 42 Ma	Calibration I

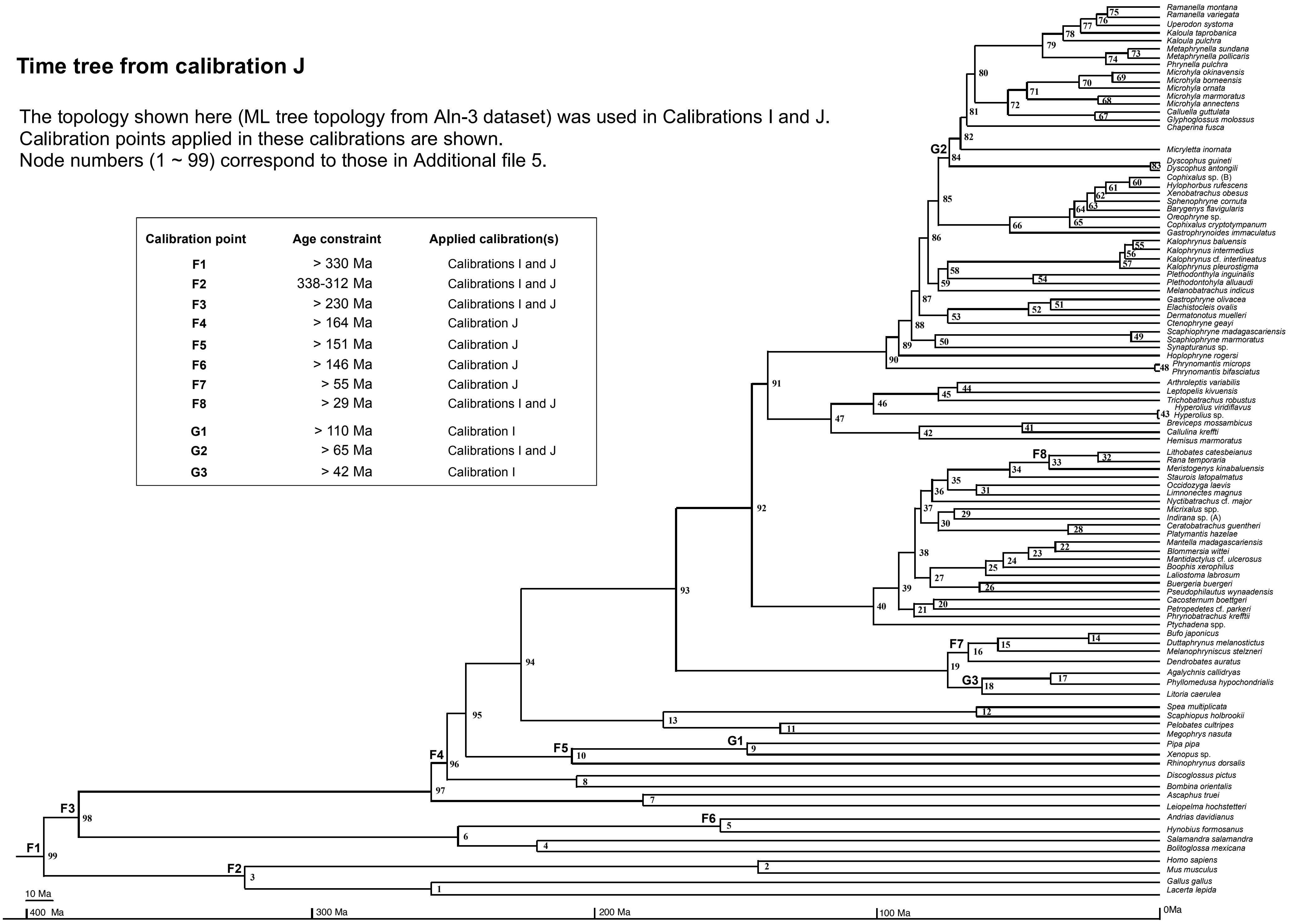

Supplement: Additional file 4 — Time trees from calibrations H and J. Time trees from the calibration H and J are shown. [file 1471-2148-11-175-S4.PDF]
